# Bisphosphonate Use and Risk of Implant Revision after Total Hip/Knee Arthroplasty: A Meta-Analysis of Observational Studies

**DOI:** 10.1371/journal.pone.0139927

**Published:** 2015-10-07

**Authors:** Songsong Teng, Chengqing Yi, Christian Krettek, Michael Jagodzinski

**Affiliations:** 1 Department of Orthopedics, Shanghai First People's Hospital, Shanghai Jiao Tong University, Shanghai, P. R. China; 2 Department of Orthopedic Trauma, Hannover Medical School, Hanover, Germany; 3 Department of Orthopedic Trauma, Agaplesion ev. Hospital Bethel, Bückeburg, Germany; Louisiana State University, UNITED STATES

## Abstract

**Objective:**

Several studies investigated the association between bisphosphonate use and the risk of implant revision after total hip or knee arthroplasty (THA or TKA); However, the findings were inconsistent. We performed this meta-analysis to evaluate the overall relative risk of such an event.

**Methods:**

We searched the PubMed, EMBASE and Cochrane library databases to identify relevant publications on April 22, 2015. To calculate the pooled risk ratios (RRs) with 95% confidential intervals (CIs), a fixed- or random-effects model was applied based on the heterogeneity across studies.

**Results:**

Three cohort studies and one case-control study were included in this meta-analysis. Compared with the bisphosphonate nonusers, the patients who used bisphosphonates for a long period of time had a significantly decreased risk of implant revision after THA/TKA (summary adjusted RR = 0.48, 95% CI: 0.38–0.61), and the summary adjusted RRs for the users who underwent THA and those who underwent TKA were 0.47 (95% CI: 0.36–0.61) and 0.45 (95% CI: 0.21–0.95), respectively.

**Conclusions:**

Long-term use of bisphosphonates is correlated with a significantly decreased risk of implant revision after THA/TKA. However, due to limited number of the included studies, the findings of the present study should be treated with caution. More well-designed studies are required to further confirm our findings.

## Introduction

Total joint arthroplasty (TJA) is a highly effective surgical procedure that contributes to excellent pain relief and great improvement in the joint function in the patients with end-stage osteoarthritis[1]. However, due to implant failure, some patients will require a revision surgery during their lifetime, which is more costly and has a worse clinical outcome than primary TJA[2]. Thus, great efforts should be made to increase the lifespan of the implants.

To our knowledge, aseptic loosening and related osteolysis are considered the most common causes for implant failure[3]. Bisphosphonates, a family of pharmacological compounds strongly inhibiting bone resorption by inactivating osteoclasts, are recommended as first line treatments for post-menopausal osteoporosis[4, 5]. It is reported that long-term use of bisphosphonates contributes to persistent antifracture and bone mineral density (BMD) increasing effects for 3–5 years after an initial 3–5 years of treatment[6]. Recently, some investigators suggest that bisphosphonates should be a treatment option for patients with osteogenesis imperfecta (OI) due to their effects on increasing BMD and reducing the risk of clinical fractures in patients with OI[7–9]. Besides, they have also been used to treat other musculoskeletal disorders, such as Paget's disease of bone[10], bone metastasis[11] and fibrous dysplasia[12]. Therefore, they may have the potential to prevent periprosthetic bone resorption. Recently, various investigations including meta-analyses and randomized controlled trials (RCTs) have demonstrated that bisphosphonates can increase periprosthetic bone mineral density (BMD) and reduce periprosthetic bone loss and prosthetic migration[13–15]. However, there are still no RCTs that determine the effect of bisphosphonates on implant survival.

Recently, several observational studies have identified the association between bisphosphonate use and risk of implant revision after total hip or knee arthroplasty (THA or TKA) [16–19], but the outcomes have been inconsistent. For example, Prieto-Alhambra et al.[16] reported that the long-term use of bisphosphonates significantly reduced the risk of revision after THA/TKA, whereas Thillemann et al. [18] did not reveal a significant association. Therefore, we performed this meta-analysis to evaluate the association between bisphosphonate use and risk of implant revision after THA/TKA from observational studies.

## Methods

### Search strategy

We systematically searched PubMed, EMBASE and the Cochrane Library databases on April 22, 2015. The search terms were (bisphosphonate) AND (total hip arthroplasty OR total hip replacement OR total knee arthroplasty OR total knee replacement OR total joint arthroplasty OR total joint replacement). The search strategies are presented in S1 Table. The reference lists of all the retrieved articles were also consulted to find potentially relevant literatures.

### Study selection criteria

The titles and abstracts were screened by two independent authors to identify the relevant studies. Then, full articles were read to employ the studies that met the following criteria: (1) used a cohort or case-control study design; (2) included participants undergoing THA or TKA; (3) used bisphosphonates before or after THA/TKA; (4) reported risk ratio (RR), hazard ratio (HR) or odds ratio (OR) with a 95% confidential interval (CI) or exhibited abundant data to calculate them; (5) English studies. Reviews, letters and conference abstracts that did not provide sufficient information were excluded.

### Data extraction and quality assessment

The following data were independently extracted for every eligible study by two authors: surname of the first author, year of publication, study location, characteristics of the patients, crude and adjusted RRs or HRs with their 95% CIs, adjusted confounders and methods used for controlling the confounders.

Two independent reviewers assessed the methodological quality of the included trials using the Newcastle-Ottawa-Scale (NOS), considering the following domains: selection of study groups (4 scores), comparability of study groups (2 scores) and assessment of outcome (cohort study, 3 scores) or exposure (case-control study, 3 scores)[20]. Investigations with scores of 0–3, 4–6, 7–9 were respectively regarded as low, moderate and high quality. Any disagreement was resolved by consensus.

### Statistical analysis

The RR was selected as the effect size to measure the association across studies. In the study that reported HR, we treated it as RR because HR is considered broadly equivalent to RR[21, 22]. The heterogeneity across studies was assessed using the Cochrane Q test and I^2^ statistic. If no significant heterogeneity (p>0.1 and I<50%) existed, an inverse-variance fixed-effects model was utilized to pool the crude and adjusted RRs with their 95% CIs among studies; Otherwise, a DerSimonian and Laird random-effects model was used to combine the data. Subgroup analysis was performed according to the adjustment for significant confounding factors. A sensitivity analysis was conducted to evaluate the stability of the results by omitting one study in each turn and pooling the data of the remaining studies. The publication bias was not assessed due to the small number of involved investigations (n<10)[23]. Data analysis was performed using RevMan Version 5.3 (Cochrane Collaboration, Oxford, UK). A p-value<0.05 was considered to be statistically significant.

## Results

### Literature search

**Fig 1:**
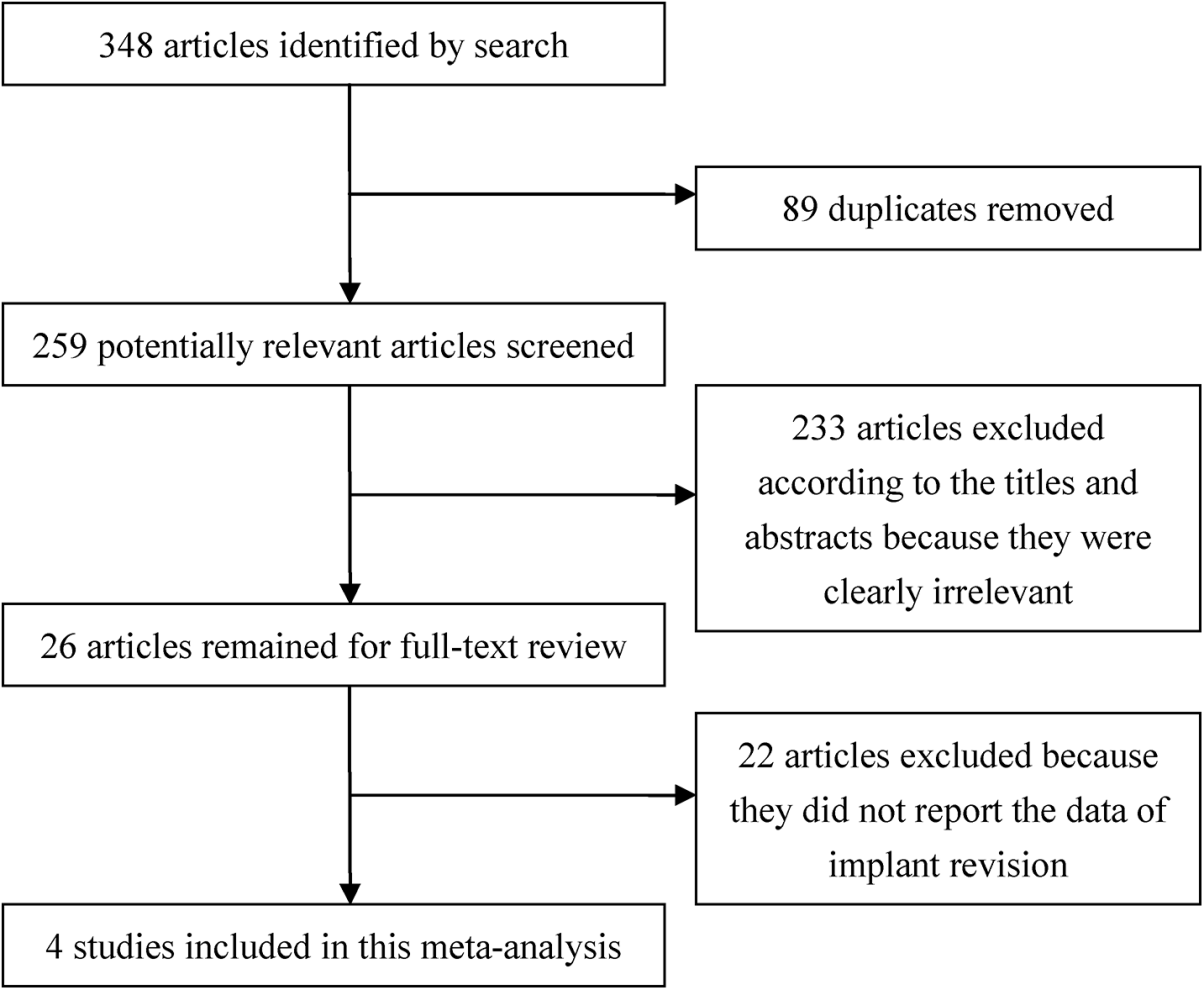
Flow chart of the selection of the publications.

The study selection process is exhibited in Fig 1. A total of 348 articles were identified by electronic searching. After 89 duplicates were removed, we screened the titles and abstracts of the remaining 259 articles. Afterwards, 233 articles were excluded because they were clearly irrelevant. After evaluating the remaining 26 publications in full, we excluded 22 articles because they did not report the data of implant revision. Finally, 4 studies were included in the present meta-analysis.

### Study characteristics

The primary characteristics of the included studies are summarized in Table 1. There are 3 cohort studies and 1 case-control study. Although 2 of the cohort studies were performed by the same group, they were based on Danish and British populations, respectively. Thus, both of them were employed in this meta-analysis. The population-based case-control study was conducted in Denmark, and a total of 1 896 subjects (cases:632; controls: 1 264) were involved. Both crude and adjusted RRs (HRs) were available in all the studies, and the primary adjusted confounding factors were age, sex, body mass index, year of primary surgery, joint replaced, use of medications other than bisphosphonates and comorbid chronic disease.

**Table 1:** Characteristics of the Included Studies in the Meta-Analysis.

Authors and year of publication	Country	Study design	Number of participants	Mean age (years)	Maximum follow-up year	Adjusted confounders	Methods used for controlling confounders	Quality score
Khatod et al., 2015 [19]	United States	Cohort	Users 2292 Nonusers 10 586	66.7	9.7	Age, sex, body mass index, American Society of Anesthesiologists score, race, diabetic status, type of implant fixation, surgeon, hospital annual mean volume, surgeon arthroplasty fellowship training **(Limited adjustment)**	Cox proportional hazards models	7
Prieto-Alhambra et al., 2014 [16]	Denmark	Cohort	Users 1558 Nonusers 8966	75.6	10	Age, sex, joint replaced (hip/knee), year of primary hip/knee replacement surgery, marital status, working status, income, number of visits to general practitioners/specialists (in the previous year), a history of fracture (other than hip fracture), osteoarthritis, comorbidity, use of medications other than bisphosphonates, use of calcium and vitamin D supplements, Charlson comorbidity index **(Good adjustment)**	Propensity score matching, Cox proportional hazards models	8
Prieto-Alhambra et al., 2011 [17]	Unite Kingdom	Cohort	Users 1912 Nonusers 40 083	69.98	15	Age, sex, body mass index, type of joint replaced (hip/knee), year of joint replacement operation, recorded diagnosis of osteoarthritis, previous fracture before surgery, use of calcium and vitamin D supplements, use of medications other than bisphosphonates, smoking status, alcohol intake, general practice deprivation score, location of surgery, comorbid conditions **(Good adjustment)**	Propensity score-adjusted regression	8
Thillemann et al., 2010 [18]	Denmark	Case-control	Cases 632 Controls 1264	>10	N/A	Age, sex, marital status, education, income, location of surgery, year of primary total hip arthroplasty, medication other than bisphosphonates, comorbid chronic disease, Charlson comorbidity index, fixation technique **(Good adjustment)**	Incidence-density matching, conditional logistic regression	8

N/A: not applicable

### Study quality and control of confounding in individual studies

The NOS was utilized to evaluate the study quality in the involved investigations. All 4 studies were high quality (S2 Table). However, because all the studies were observational, particular care was needed to examine confounding. Among them, matching was used to control the confounding in 2 studies during the study design stage, and multivariable analysis was performed in all the studies. Three studies were judged to have good control of confounding, and one study was judged to have limited control of confounding. However, in 3 of the 4 included studies, confounding by indications was likely. Baseline characteristics showed that bisphosphonate users had more comorbidities than nonusers. Moreover, bisphosphonate users were also more likely to be the users of calcium/vitamin D supplements. Residual confounders, such as smoking, physical activity, design of implant, can also contribute to the risk of bias

### Implant revision after THA/TKA

**Fig 2:**
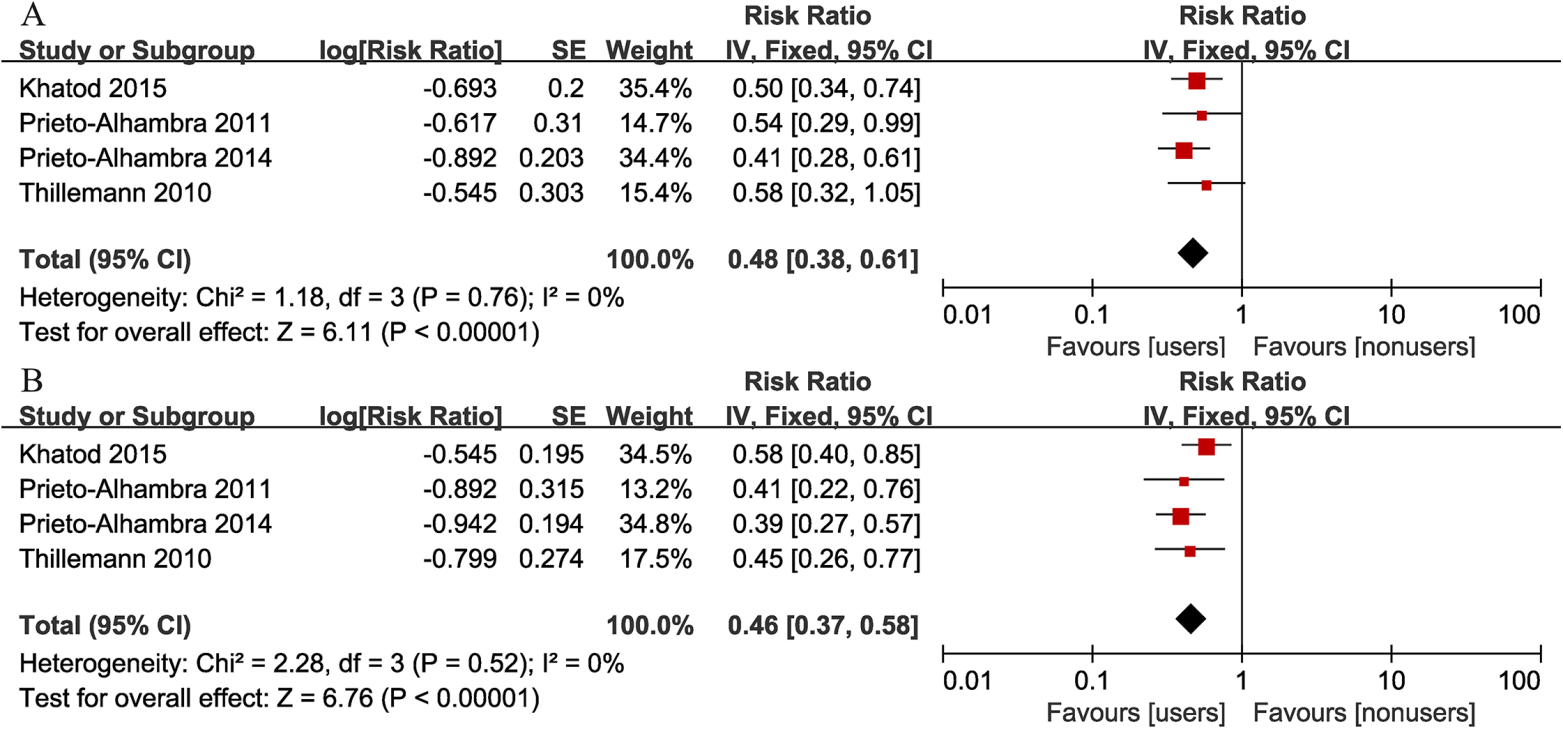
Forest plot of the association between bisphosphonate use and risk of implant revision after total hip/knee arthroplasty (A: adjusted RR; B: crude RR).

Fig 2 shows a forest plot presenting the association between bisphosphonate use and risk of all-cause revisions after THA/TKA. No heterogeneity was found across studies (I^2^ = 0%). Compared with nonusers, bisphosphonate users had a significantly decreased risk of revision surgery (summary adjusted RR = 0.48, 95% CI: 0.38–0.61). Sensitivity analysis was performed by omitting one study in each turn, analyzing the combined RRs for the rest studies. The overall adjusted RRs, ranging from 0.47 (95% CI: 0.35–0.63) to 0.53 (95% CI: 0.39–0.70), did not exhibit significant variation, indicating the stability of the outcome. When using crude data, the association was also found (summary crude RR = 0.46, 95% CI: 0.37–0.58), and sensitivity analysis revealed no significant variation.

### Subgroup analysis

Table 2 presents the outcomes of subgroup analysis according to the adjustment for significant confounding factors including age, gender, body mass index, smoking, type of implant fixation, year of primary surgery, location of surgery, use of calcium and vitamin D, use of medications other than bisphosphonates, number of visits to general practitioners and specialists, cormobidities. and adequacy of confounding control. Generally, for the results by fixed-effects model, the pooled RRs were similar to the overall RRs, and the significant association between bisphosphonate use and risk of implant revision after THA/TKA remained. However, we found that the study that controlled the number of visits to general practitioners/specialists had a lower adjusted RR (0.41; 95% CI: 0.28, 0.61) than the studies that did not control it (adjusted RR = 0.53; 95% CI: 0.39–0.70), indicating that number of visits to general practitioners/specialists is a significant confounding factor that have to be adjusted because the patients who visit the practitioners/specialists more can undergo a better therapy.

**Table 2:** Subgroup analysis according to the adjustment for significant confounding factors.

Adjustment for confounding	Level	No. of studies	I^2^ (crude)	Crude RR (95% CI)	I^2^ (adjusted)	Adjusted RR (95% CI)
Age>40	Yes	3	12%	0.46 [0.36, 0.59]	0	0.47 [0.36, 0.60]
	No	1	0	0.45 [0.26, 0.77]	0	0.58 [0.32, 1.05]
Female>60%	Yes	2	0	0.41 [0.30, 0.56]	0	0.46 [0.33, 0.64]
	No	2	0	0.53 [0.38, 0.73]	0	0.51 [0.37, 0.71]
Body mass index	Yes	2	0	0.53 [0.38, 0.73]	0	0.51 [0.37, 0.71]
	No	2	0	0.41 [0.30, 0.56]	0	0.46 [0.33, 0.64]
Smoking	Yes	1	0	0.41 [0.22, 0.76]	0	0.54 [0.29, 0.99]
	No	3	5%	0.47 [0.37, 0.60]	0	0.47 [0.37, 0.61]
Use of calcium and vitamin D	Yes	2	0	0.40 [0.29, 0.55]	0	0.45 [0.32, 0.62]
	No	2	0	0.53 [0.39, 0.73]	0	0.52 [0.38, 0.73]
Use of medications other than bisphosphonates	Yes	3	0	0.41 [0.31, 0.54]	0	0.47 [0.35, 0.63]
	No	1	0	0.58 [0.40, 0.85]	0	0.50 [0.34, 0.74]
Number of visits to general practitioners/specialists	Yes	1	0	0.39 [0.27, 0.57]	0	0.41 [0.28, 0.61]
	No	3	0	0.50 [0.38, 0.67]	0	0.53 [0.39, 0.70]
Year of primary surgery	Yes	3	0	0.41 [0.31, 0.54]	0	0.47 [0.35, 0.63]
	No	1	0	0.58 [0.40, 0.85]	0	0.50 [0.34, 0.74]
Location of surgery	Yes	2	0	0.43 [0.29, 0.65]	0	0.56 [0.37, 0.86]
	No	2	52%	0.48 [0.32, 0.70]	0	0.45 [0.34, 0.60]
Type of implant fixation	Yes	2	0	0.53 [0.39, 0.73]	0	0.52 [0.38, 0.73]
	No	2	0	0.40 [0.29, 0.55]	0	0.45 [0.32, 0.62]
Comorbidities	Yes	3	0	0.41 [0.31, 0.54]	0	0.47 [0.35, 0.63]
	No	1	0	0.58 [0.40, 0.85]	0	0.50 [0.34, 0.74]
Control of confounding	Good	3	0	0.41 [0.31, 0.54]	0	0.47 [0.35, 0.63]
	Limited	1	0	0.58 [0.40, 0.85]	0	0.50 [0.34, 0.74]

### Implant revision after TKA

**Fig 3:**
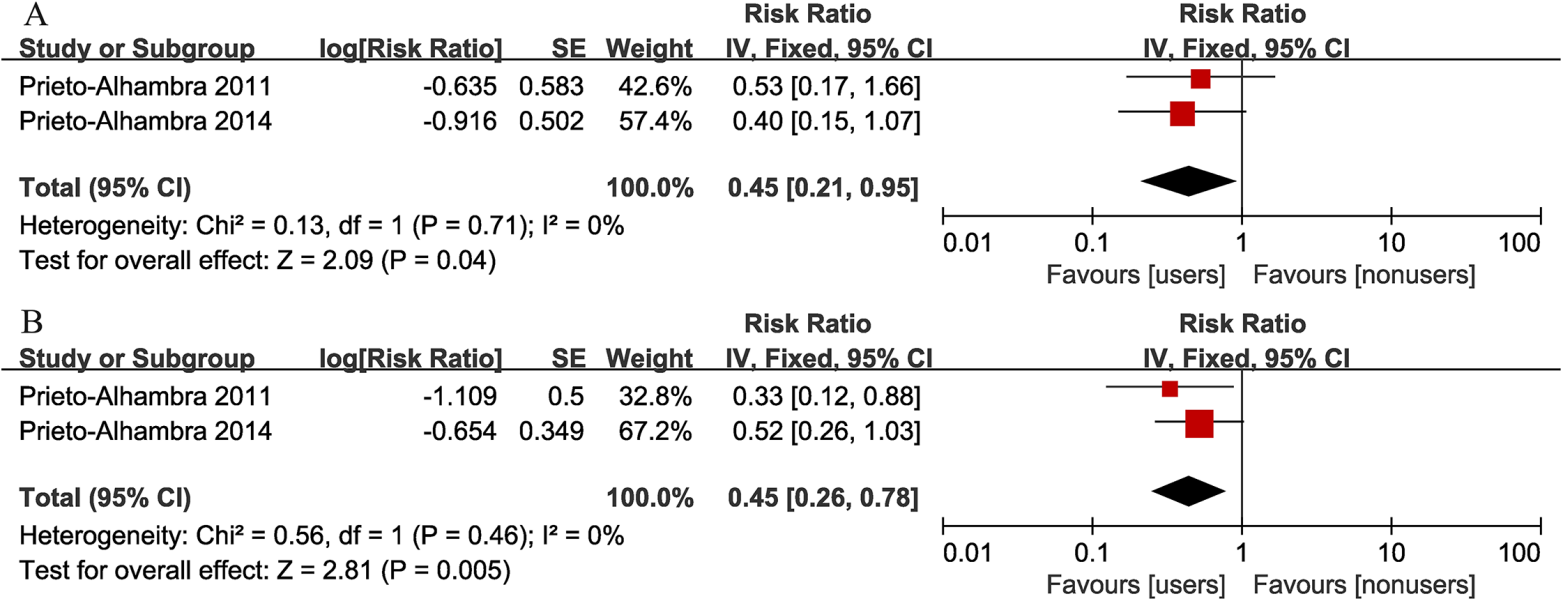
Forest plot of the association between bisphosphonate use and risk of implant revision after total knee arthroplasty (A: adjusted RR; B: crude RR).

The revision risk of TKA was documented in 2 studies. There was no heterogeneity between the 2 studies (I^2^ = 0%). We found a significantly reduced risk of revision surgery in the bisphosphonate users than in nonusers (summary crude RR = 0.45, 95% CI: 0.26–0.78; summary adjusted RR = 0.45, 95%: 0.21–0.95) (Fig 3).

### Implant revision after THA

**Fig 4:**
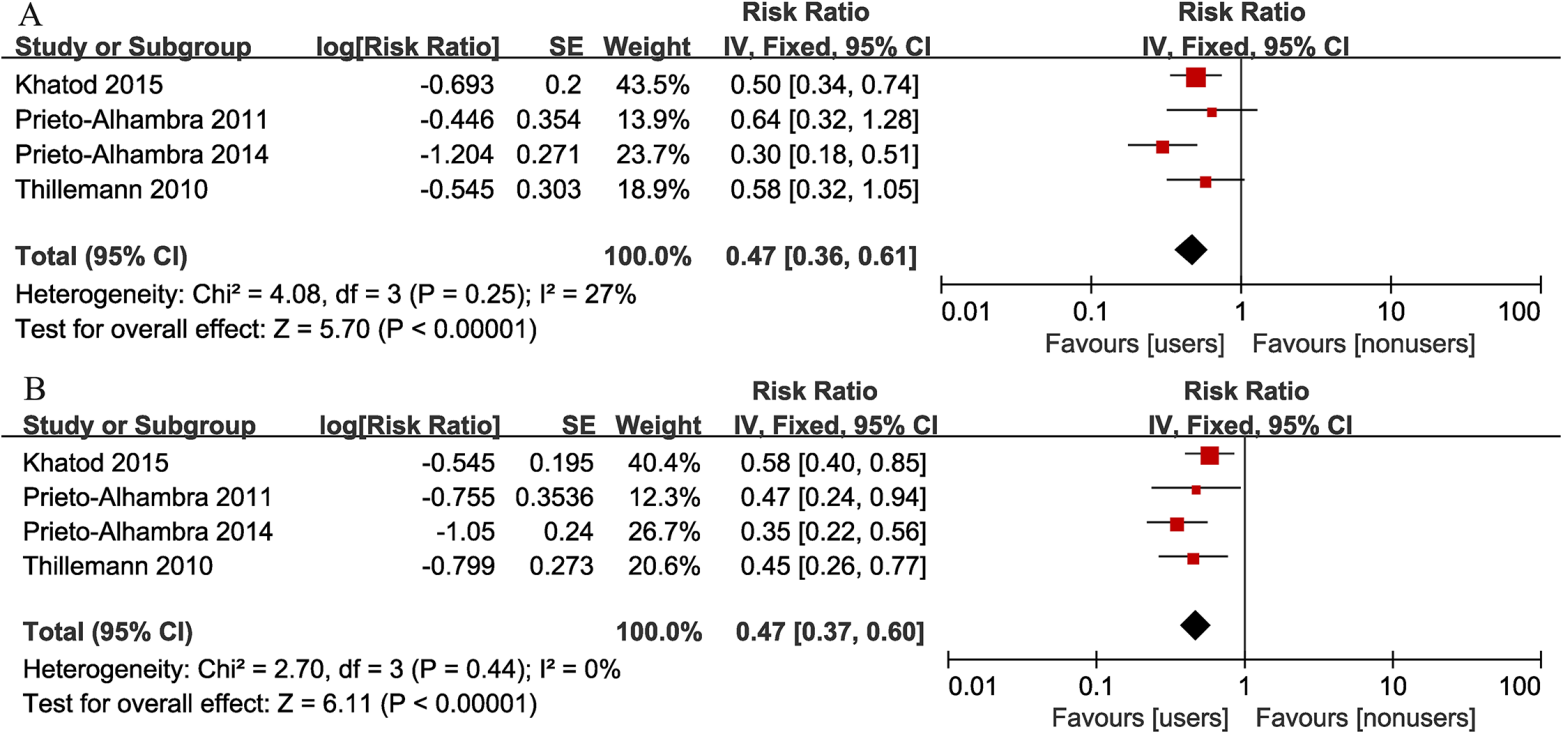
Forest plot of the association between bisphosphonate use and risk of implant revision after total hip arthroplasty (A: adjusted RR; B: crude RR).

The revision risk after THA was mentioned in all 4 studies. There was moderate heterogeneity across studies (I^2^ = 27%, p = 0.25). Meta-analysis using a fixed-effects model suggested bisphosphonate users had a significantly decreased risk of revisions than nonusers (summary adjusted RR = 0.47, 95% CI: 0.36–0.61) (Fig 4). We also performed a meta-analysis using a random-effects model due to the moderate heterogeneity with a I^2^ = 27%. The summary adjusted RR was 0.47 (95% CI: 0.35–0.65), reconfirming the significantly reduced risk in bisphosphonate users. Moreover, sensitivity analysis was carried out based on the method above. The pooled RRs by excluding any of the studies, ranging from 0.44 (95% CI: 0.31–0.64) to 0.54 (95% CI: 0.4–0.73), did not vary substantially, demonstrating the stability of the present outcome. The pooled crude RR is also significant (summary crude RR = 0.47, 95% CI:0.37–0.60) with no heterogeneity (I^2^ = 0), and sensitivity analysis was conducted, yielding similar results.

### Preoperative bisphosphonate use and risk of revision after THA/TKA

**Fig 5:**
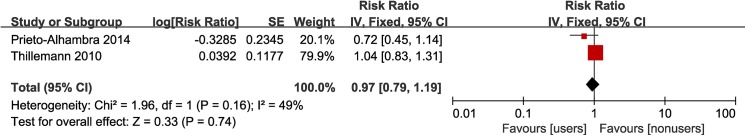
Forest plot of the association between preoperative bisphosphonate use and risk of implant revision after total hip/knee arthroplasty.

Two studies analyzed the association between preoperative bisphosphonate use and risk of revision after primary TJA. Moderate heterogeneity existed between two studies (I^2^ = 49%, p = 0.16). Meta-analysis using a fixed-effects model suggested that the preoperative use of bisphosphonates did not reduce the risk of revision after THA/TKA (summary adjusted RR = 0.97, 95% CI: 0.79–1.19) (Fig 5), which was consistent with the result of the meta-analysis using a random-effects model (summary adjusted RR = 0.92, 95% CI: 0.65–1.29).

## Discussion

In our meta-analysis of 3 cohort studies and 1 case-control study, bisphosphonate users had a significantly lower risk of implant revision after THA/TKA than nonusers when we pooled either crude RRs or adjusted RRs. The combined effect size for the patients who underwent THA was similar to that for those who underwent TKA.

To date, a variety of causes that contribute to implant failures after TJA have been identified[24–28]. A complication-based analysis using worldwide arthroplasty registers demonstrated that aseptic loosening was the most common cause for revisions both in THA (55.2%) and TKA (29.8%) [29]. Recently, a meta-analysis of RCTs suggested a protective effect of bisphosphonates on preventing periprosthetic bone loss after TJA[15], which could persist 18 to 72 months after discontinuation of bisphosphonates, and the second and third generation of bisphosphonates had a better efficacy than the first generation. Some investigators also reported that postoperative use of bisphosphonates significantly decreased implant migration after TJA[30, 31]. Recently, Sorenson et al. [32] demonstrated that bisphosphonate treatment enhanced early stability of revision joint replacements without preventing new bone formation. Furthermore, the use of bisphosphonates could increase the periprosthetic BMD around the whole stem and the cup by 2.4% and 7.1%, respectively, in the patients affected by periprosthetic osteolysis after THA, and there was a great improvement in pain and function, indicating that bisphosphonate treatment prevents wear debris mediated osteolysis[33]. All of these evidences suggest that the use of bisphosphonates after TJA may be beneficial for the prevention of osteolysis and stress shielding around the implants and finally reducing the prosthetic failure and revision surgery[19].

Among the studies included in the present meta-analysis, all 3 cohort studies reported that long-term use of bisphosphonates significantly decreased the risk of implant revision[16, 17, 19], whereas the case-control study showed a non-significant association, but the authors proposed that long-term use of bisphosphonate had a reduced risk of implant revision compared to short-term use[18], which is consistent with the outcomes in the study by Curtis et al.[34]. They demonstrated that short-term bisphosphonate treatment did not benefit bone strength. Because the authors of the cohort studies only defined the patients who underwent at least 182 days or 6 months of bisphosphonate treatment as bisphosphonate users, we extracted the crude and adjusted RR of revision for the patients who underwent more than 240 day treatment of bisphosphonates in the study conducted by Thillemann et al. [18] for meta-analysis. Our current investigation demonstrated that bisphosphonate users had a 52% lower risk of implant revision than nonusers. Although there was a higher effect estimate for the patients who had THA than those who had TKA in the study performed by Prieto-Alhambra et al. [16] in 2014, the pooled RRs of this meta-analysis showed a similar effect size for them, indicating bisphosphonates may have a similar effect on preventing the failures of THA and TKA. Besides the reduction of the risk of revision, use of bisphosphonates also significantly reduced the increased risk of fracture after THA/TKA[35]. Therefore, the use of bisphosphonates has a promising application potential for reducing implant failure and extending implant survival after THA/TKA. More importantly, this treatment is relatively costless and easy to handle, and the patients may be willing to accept this therapy, especially for the elderly, whose physical conditions cannot allow them to undergo a revision surgery. However, all the studies included in the meta-analysis were cohort studies. RCTs are required to evaluate the effectiveness of bisphosphonate treatment on the extension of implant survival.

Interestingly, in the present meta-analysis, we found that the patients who started the treatment of bisphosphonates preoperatively didn't have a lower risk of revision surgery compared to nonusers. It is still not very clear why preoperative bisphosphonate therapy does not benefit implant integration. Prieto-Alhambra et al. [16] inferred that preoperative use of bisphosphonates could reduce bone remodeling, which might prevent the regeneration of the underlying bone and harm osteointegration. In contrast, postoperative bisphosphonate treatment favors the reduction of inflammation-mediated osteolysis around implant and effectively inhibits aseptic loosening of prosthesis after TJA. In the study by Thillemann et al. [18], the authors found that the length of bisphosphonate use after THA was inversely correlated with the risk of implant revision, and the patients who used the bisphosphonates for a short-time period had a significantly increased risk of implant revision due to deep infection, which was not observed in the patients undergoing a long-time treatment of bisphosphonates after THA. All these findings demonstrate that unreasonable use of bisphosphonates after TJA may lead to implant failure. In addition, long-term use of bisphosphonates may result in some severe side effects, such as esophageal cancer, osteonecrosis jaw and atrial fibrillation[36]. Some studies also demonstrated the progression of necrosis of the jaw even after bisphosphonate interruption[37]. To date, no consensus has been reached on the time limitation of bisphosphonate use. The optimal dose frequency, doze size and duration of bisphosphonate therapy remain to be determined to avoid severe adverse effects without reducing the efficacy. Nevertheless, large prospective cohort studies with adequate duration are necessary to further confirm this finding due to the moderate heterogeneity and limited number of the included studies (n = 2).

There are several limitations in this study. First, the included publications were observational studies, which cannot reveal causality, but provide information on associations. Compared with RCTs, observational studies have a lower level of evidence. Lack of more RCTs indicates our meta-analysis is based on rather limited evidence, which limits the quality of the meta-analysis. Second, the quality of the included studies varied. Although all the included studies controlled the primary confounders, the residual confounding, such as smoking, BMD of the patients, type of implants and amount of exercise after surgery, may still affect the association. For example, in one of our previous studies, we found that smoking may significantly increase the risk of all-cause revisions after THA[38]. This critical factor was not considered in most studies, and physical activity is a known risk factor for the aseptic loosening of the implants[39], whereas none of the authors takes it into account, which may result in the miscalculation of the outcomes. In our subgroup analysis, we found that number of visits to practitioners/specialists was a significant confounding required to be controlled. According to the baseline characteristics, confounding by indications may exist, and the bisphosphonate users were more likely to intake calcium/vitamin D supplements, which may also affect the overall RR. Moreover, moderate heterogeneity existed between studies. However, sensitivity analyses suggested that the outcomes of this meta-analysis were rather robust. Third, the small number of studies is a main limitation of this study, which may lead to a high risk of bias. Therefore, more prospective studies are needed to confirm the association.

## Conclusions

The present meta-analysis suggests that long-term bisphosphonate use is associated with a significantly reduced risk of implant revision after THA/TKA. However, because of the limited number and varied quality of the included studies, these findings should be treated with caution, and more well-designed investigations, like RCTs, are required to validate our findings.

## Supporting Information

S1 PRISMA Checklist (DOC)

S1 Table Search strategy for PubMed on April 22, 2015. (DOC)

S2 Table Methodological quality of the studies included in the meta-analysis. (DOC)
