# Supplementary material for: Bisphosphonate Use and Risk of Implant Revision after Total Hip/Knee Arthroplasty: A Meta-Analysis of Observational Studies
Source: PLoS One. 2015 Oct 7;10(10):e0139927. doi: 10.1371/journal.pone.0139927 (PMC4596810; doi:10.1371/journal.pone.0139927)
Supplement: S1 Table — (DOC) [file pone.0139927.s002.doc]

**S1 Table Search Strategy for PubMed on April 22, 2015**

| Search strategy | Search terms |
| --- | --- |
| #1 | total hip arthroplasty |
| #2 | total hip replacement |
| #3 | total knee arthroplasty |
| #4 | total knee replacement |
| #5 | total joint arthroplasty |
| #6 | total joint replacement |
| #7 #1 OR #2 OR #3 OR #4 OR #5 OR #6 |  |
| #8 | bisphosphonate |
| #9 #7 AND #8 |  |
