# Supplementary material for: Bisphosphonate Use and Risk of Implant Revision after Total Hip/Knee Arthroplasty: A Meta-Analysis of Observational Studies
Source: PLoS One. 2015 Oct 7;10(10):e0139927. doi: 10.1371/journal.pone.0139927 (PMC4596810; doi:10.1371/journal.pone.0139927)
Supplement: S2 Table — (DOC) [file pone.0139927.s003.doc]

**S2 Table.** Methodological quality of the studies included in the meta-analysis

| **Study** | **Selection** |  |  |  | **Comparability** | **Outcome/Exposure** |  |  | **Total scores** |
| --- | --- | --- | --- | --- | --- | --- | --- | --- | --- |
|  | **Representativeness of the exposed cohort/cases** | **Selection of the non-exposed cohort/controls** | **Ascertainment of exposure/definition of controls** | **Outcome of interest not present at start of study/adequacy of case definition** | **Control for important factors or additional factors** | **Outcome assessment/** **Ascertainment of exposure** | **Follow-up long enough for outcomes to occur/ Same method of ascertainment for cases and controls** | **Adequacy of follow-up of cohorts/** **Non-Response rate** |  |
| Khatod et al.,2015 [19] | ☆ | ☆ | ☆ | - | ☆ | ☆ | ☆ | ☆ | 7 |
| Prieto-Alhambra et al., 2014 [16] | ☆ | ☆ | ☆ | - | ☆☆ | ☆ | ☆ | ☆ | 8 |
| Prieto-Alhambra et al., 2011 [17] | ☆ | ☆ | ☆ | - | ☆☆ | ☆ | ☆ | ☆ | 8 |
| Thillemann et al., 2010 [18] | ☆ | ☆ | ☆ | - | ☆☆ | ☆ | ☆ | ☆ | 8 |
